# Bisphenol A Induces Accelerated Cell Aging in Murine Endothelium

**DOI:** 10.3390/biom11101429

**Published:** 2021-09-29

**Authors:** Rafael Moreno-Gómez-Toledano, Sandra Sánchez-Esteban, Alberto Cook, Marta Mínguez-Moratinos, Rafael Ramírez-Carracedo, Paula Reventún, María Delgado-Marín, Ricardo J. Bosch, Marta Saura

**Affiliations:** 1 Universidad de Alcalá, Systems Biology Department, IRYCIS, 28772 Alcalá de Henares, Spain; rafael.moreno@uah.es (R.M.-G.-T.); sandra.sancheze@uah.es (S.S.-E.); alberto.cook@edu.uah.es (A.C.); marta.minguez.moratinos@gmail.com (M.M.-M.); prevent1@jhmi.edu (P.R.); mariadm.2898@gmail.com (M.D.-M.); ricardoj.bosch@uah.es (R.J.B.); 2 Cardiology Research Unit, Universidad Francisco de Vitoria, 28223 Madrid, Spain; rrcarracedo@hotmail.com

**Keywords:** bisphenol A, murine aortic endothelial cell, senescence, unfolding protein response

## Abstract

Bisphenol A (BPA) is a widespread endocrine disruptor affecting many organs and systems. Previous work in our laboratory demonstrated that BPA could induce death due to necroptosis in murine aortic endothelial cells (MAECs). This work aims to evaluate the possible involvement of BPA-induced senescence mechanisms in endothelial cells. The β-Gal assays showed interesting differences in cell senescence at relatively low doses (100 nM and 5 µM). Western blots confirmed that proteins involved in senescence mechanisms, p16 and p21, were overexpressed in the presence of BPA. In addition, the UPR (unfolding protein response) system, which is part of the senescent phenotype, was also explored by Western blot and qPCR, confirming the involvement of the PERK-ATF4-CHOP pathway (related to pathological processes). The endothelium of mice treated with BPA showed an evident increase in the expression of the proteins p16, p21, and CHOP, confirming the results observed in cells. Our results demonstrate that oxidative stress induced by BPA leads to UPR activation and senescence since pretreatment with N-acetylcysteine (NAC) in BPA-treated cells reduced the percentage of senescent cells prevented the overexpression of proteins related to BPA-induced senescence and reduced the activation of the UPR system. The results suggest that BPA participates actively in accelerated cell aging mechanisms, affecting the vascular endothelium and promoting cardiovascular diseases.

## 1. Introduction

Bisphenol A (BPA) is a phenolic-type molecule widely used in the manufacture of plastics. Its ability to improve the properties of plastics and its role as a monomer in synthesizing polycarbonates and epoxy resins have made it an element present in many everyday objects [1,2]. BPA has become an essential element for many global industries, from food containers, toys, and clothing to electronic devices and medical-surgical supplies [1,3,4,5,6,7]. Its production and demand increase every year, and the trend is expected to continue upward in the coming years [8].

BPA is classified as an endocrine disruptor or xenoestrogen due to its ability to bind to estrogen receptors [9]. For this reason, numerous studies are exploring its possible effects on both the female and male genitourinary systems [10,11,12,13]. However, in the last two decades, evidence correlates BPA with other types of pathologies such as diabetes, obesity, and even cognitive and behavioral disorders [14,15,16,17]. Our group has recently found new evidence that positions BPA as a possible environmental factor promoting nephro-vascular pathologies [18,19,20,21]. Currently, few studies explore the cardiovascular system, but there are population studies that correlate high concentrations of urinary BPA with an increased risk of developing arterial hypertension [22,23].

A process highly implicated in the development of cardiovascular pathologies is aging and cellular senescence [24]. Cellular senescence is a process that can be considered a hallmark of aging [25] and is characterized by the irreversible arrest of the cell cycle in the presence of different inducing factors, such as telomere dysfunctions, oxidative stress, activation of oncogenes, or cell damage [26]. Senescence is frequently called quiescence, although there are notable differences between the two processes at the morphological and molecular levels [27]. The p53/p21 and p16/pRB pathways are classical pathways regulating cellular senescence [26]. There is substantial evidence that supports the close relationship between cellular senescence and cardiovascular disorders. In humans, significant increases in the expression of senescence proteins have been observed in endothelial cells of aged sedentary individuals, compared with aged exercising adults (57–60 years old) [24]. Furthermore, there is evidence that BPA could be involved in the acceleration of cellular aging in different cell lines [28,29], species [30], as well as in human studies [31]. We previously reported that BPA can induce arterial hypertension through angiotensin II (AngII)-mediated eNOS uncoupling [18]. Importantly, AngII stimulation recapitulates the senescent characteristics in vascular cells, which may contribute to the acceleration of atherosclerosis [32,33]. Aged human [34] and murine [35,36] arterial endothelial cells as well as EPCs [37,38] also show heightened levels of ROS partially due to activation of NADPH oxidase, which further uncouples eNOS [34,39,40]. All of the above led us to formulate the hypothesis that BPA could induce endothelial cell senescence.

A fundamental component activated in response to stress within the senescent phenotype is the unfolding protein response (UPR). The activation of proteins of the UPR system in response to senescence-inducing stimuli has been observed in different cell lines, and the inhibition of some of these proteins can modify the percentage of SA-β-galactosidase-positive cells [41]. The UPR system has three main signaling pathways, initiated by the ATF6, IRE1, and PERK stress sensors. Activation of each sensor activates the transcription factors ATF6 (N), XBP1, and ATF4, respectively. These factors are capable of increasing the folding capacity of the endoplasmic reticulum [42]. An important difference between the signaling pathways is that the PERK-ATF4-CHOP pathway is related to myocardial ischemia, hypertension, cardiac atrophy, hypertrophy, or even vascular calcification [43,44,45,46,47], while the IRE1 and ATF6 pathways may have cardioprotective effects [43,48].

Classically, the PERK-ATF4-CHOP signaling pathway has been associated with cell apoptosis [49,50,51,52]. Other authors have observed that the treatment of BPA at cytotoxic concentrations in non-parenchymal hepatocytes mice (100 µM) [51] or mouse spermatocytes GC-2 cells (20–80 µM) [52] induced a significant increase in apoptotic processes related to ER stress. However, there is evidence in the literature that the reduction in the PERK-ATF4-CHOP pathway can reduce the number of senescent cells in certain cell lines, such as melanocytes [41]. It has even been observed that the overexpression of CHOP in senescent cells does not lead to cell death [53].

The present work aims to explore the role of BPA in endothelial cell senescence, exploring the involvement of the unfolding protein response. Thus, BPA may contribute to cardiovascular diseases by activating this mechanism of accelerated cellular aging.

## 2. Materials and Methods

### 2.1. Cell Culture

Murine aortic endothelial cells (MAECs) were isolated from mouse aorta, as previously reported [19,54]. Briefly, the aortas were sectioned a deposited in Matrigel solution and fed with fresh growth medium for seven days (DMEM/HAM’s medium, 20% FBS, 0.05 mg/mL penicillin/streptomycin, and 2.5 μg/mL amphotericin). The tissue was removed, and 500 μL of cell recovery solution was added to each culture. The solution was centrifuged at 4 °C, resuspended in 4 mL of growing medium, and plated. MAECs were selected by their ability to express the intercellular adhesion molecule-2 (ICAM-2) protein and purified with a flow cytometry cell sorter (DAKO, Carpinteria, CA, USA). Purification was verified by confocal microscopy of MAECs double-stained with Von Willebrand factor antibodies.

MAEC were cultured with Dulbecco’s Modified Eagle’s Medium (DMEM/F12), supplemented with 0.05 mg/mL penicillin/streptomycin, 2.5 μg/mL amphotericin, and 10% fetal bovine serum (Gibco, Waltham, MA, USA) in a humidified CO_2_ incubator with 5% CO_2_ at 37 °C. MAEC were used between passage 4 and 9. BPA concentrations between 1 nM and 100 µM were used to delimit the cytotoxicity on the MTT assay. Subsequently, in the senescence test, doses lower than cytotoxic in the range of 1 nM–5 µM were used (5 µM is half of the maximum concentration at which no cytotoxic effects were observed). The two concentrations at which the highest effects on cell senescence were observed (100 nM and 5 µM) were used for Western blot and qPCR. Finally, in the approach with the antioxidant N-Acetyl-l-cysteine (NAC) (Sigma, San Luis, MO, USA), a concentration commonly used in the academic literature was used, 5 mM two hours before treatment with BPA [55,56].

### 2.2. Animal Model

Wild-type CD1 mice were purchased from Charles River (Wilmington, MA, USA) and housed in our animal facilities with four mice/cage located in isolated rooms. All animal procedures were approved by the University of Alcala Animal Care Committee and Autonomous Community of Madrid) and conformed to the EU directive regarding protecting animals used for experimental and other scientific purposes (enacted under Spanish law 1201/2005).Three-month-old CD1 male mice (~40 g weight) were distributed into two groups: control and BPA. Control treatment consisted of an equivalent volume of ethanol (final concentration 0.01%) in drinking water. Ethanol dissolved-BPA was added to the drinking water at a final concentration of 150 µg/mL. This dose of BPA is equivalent to 25 mg/kg; that is, half of the concentration used by the European Food Safety Authority as a reference in the calculation of the tolerable daily intake [1] and also the U.S. National Toxicology Program defines doses less than 50 mg/kg/day as “low dose” [57]. Furthermore, in the CLARITY-BPA study (Consortium Linking Academic and Regulatory Insights on Bisphenol A Toxicity), one of the largest animal studies conducted in the context of BPA, the authors stated, “BPA did not elicit adverse effects in the in-life or terminal endpoints monitored in either sex below 25,000 μg/kg bw/day” [58]. However, other authors suggest that some results described in this study show non-monotonic effects, reaffirming the need to study the effects of BPA at low doses [59].

Mice received BPA or vehicle in the drinking water for 8 weeks. Mice were given free access to water and drank approximately 5 mL/day/mouse [18]. At the end of the experiment, animals were sacrificed, and aortas were collected and stored for posterior Western blot and immunofluorescence.

### 2.3. MTT Cell Viability Assay

MAEC were then seeded into 24 well plates (1500 cells/well) in complete medium. After overnight incubation, the medium was removed, and 1 mL of growth culture containing a series of different concentrations of BPA ranging from 0 (control) to 100 μM was added during 5 days. After BPA treatment, 100 µL of MTT (5 mg/mL) were added to each well in 900 µL of the medium, and the plates were incubated for 3 h at 37 °C. Then, DMSO (Sigma, San Luis, MO, USA) was added to solubilize the formazan crystals. The absorbance was measured at a test wavelength of 570 nm [60].

### 2.4. Senescence-Associated β-Galactosidase Assay

Senescence-associated β-Galactosidase (β-Gal) staining was performed on mouse aortic endothelial cells (MAEC) using a senescence detection kit (Abcam) according to the manufacturer’s protocol. Briefly, cells were fixed and incubated with staining solution mix for 12 h at 37 °C. After that time, the cells were photographed under the microscope (Nikon DIAPHOT 300, Tokyo, Japan). Eight photographs were taken per well in different areas using the 10× objective. Afterward, a count of senescent and total cells was performed, and the data were analyzed.

### 2.5. Western Blot

Protein lysates were immunoblotted as previously described [61]. Total protein was separated in SDS-polyacrylamide gel electrophoresis and transferred to a PDVF membrane. For protein detection, blocked membranes were incubated with specific antibodies, washed, and incubated with a secondary antibody. Immunoreactive bands were visualized with the SuperSignal detection system Pierce™ ECL Western Blotting Substrate (Thermo Fisher Scientific, Waltham, MA, USA). Primary antibodies used: p16 from Abcam (reference ab51243; 1:1000), p21 from eBioScience (reference 14-6715-81; 1:1000), CHOP from Cell Signaling (reference 5554S; 1:500), XPB1 from Sigma (reference SAB2102720; 1:500) and β-Actin from Sigma (reference A2066; 1:1000).

### 2.6. Protein Oxidation

Protein oxidation detected by reaction with 2,4-dinitrophenyl hydrazine (DNP) using an OxyBlot™ Protein Oxidation Detection Kit (Sigma, San Luis, MO, USA). Briefly, samples were denatured by SDS, and the carbonyl groups in the protein side chains were derivatized to DNP-hydrazone by reaction with DNPH. The proteins were electrophoresed on an SDS-PAGE gel and followed by immunoblotting of the anti-DNP antibody (1:150). The same membrane was incubated with the anti-b-actin antibody for loading control. For densitometric analysis, the proteins migrating between 97 and 68 KDa were analyzed.

### 2.7. qPCR

Total RNA was extracted with TRIzol reagent from Invitrogen Corporation (Carlsbad, CA, USA) following the manufacturer´s instructions. First-strand cDNA was synthesized from 2 μg of total RNA in a 20 μL reaction mixture using the High-Capacity cDNA reverse transcription kit, and the qPCR reaction was performed with SYBR select master mix both of Life technologies (Carlsbad, CA, USA). The qPCR conditions were standard cycling mode (primer Tm ≥ 60 °C) first UDG activation 50 °C 2 min, then AmpliTaq^®^ DNA polymerase, UP activation 95 °C 2 min, denature 95 °C 15 s and anneal/Extend 60 °C 1 min. The following primers were used: CHOP forward 5′ CAG GAG AAC GAG CGG AAA GTG G 3′, CHOP reverse 5′ TGC TGG GTA CAC TTC CGG AGA G 3′, ATF-6 forward 5′ TTC GAG GCT GGG TTC ATA G 3′, ATF-6 reverse 5′ GGG AGG CGT AAT ACA CTT 3′, PERK forward 5′ ATG CAC AGG GAC CTC AAG 3′, PERK reverse 5′ CTG CTC TGG GCT CAT GTA TAG 3′, IRE-1α forward 5′ AAG ATG GAC TGG CGG GAG AAC IRE-1α reverse 5′ GGG AAG CGG GAA GTG AAG TAG 3′, Actin forward 5′ CGA TGC CCT GAG GCT CTT T 3′, Actin reverse 5′ TGG ATG CCA CAG GAT TCC A 3′.

### 2.8. Immunohistochemistry

Aorta arteries were fixed in a 10% formalin solution, dehydrated in ethanol, and then embedded in paraffin as previously described [62]. Tissue sections (5 μm) were obtained in a microtome, were deparaffinized, rehydrated, and stained. Samples were boiled in retrieval buffer for 20 min after xylene deparaffinization. A Mouse- and Rabbit-Specific HRP/DAB (ABC) Detection IHC kit (Abcam, Cambrige, UK) was used according to the manufacturer’s protocol, and antibody incubation was overnight at 4 °C. Sections were counterstained with Carazzi hematoxylin, dehydrated, and mounted with DPX (Casa Alvarez, Madrid, Spain). Images obtained of at least five different animals per condition were taken for data quantification using a bright-field microscope (Eclipse 50i; Nikon, Tokyo, Japan). Primary antibodies used in immunohistochemistry: p16 from Abcam (reference ab51243; 1:50), p21 from eBioScience (reference 14-6715-81; 1:50) and CHOP from Cell Signaling (reference 5554S; 1:50).

### 2.9. Confocal Microscopy

Slides containing tissue sections were incubated with the primary antibodies overnight 4  °C. After washing with PBS, the slides were incubated with FITC, Alexa-488, or Alexa-647-conjugated secondary antibodies for 1 hour at room temperature. Isolectin IB4 labeled with FITC was used as a specific marker of vascular endothelium. Nuclei were stained with Hoechst. Images were taken for data quantification using a Leica TCS SP5 confocal microscope (UAH-NANBIOSIS-CIBER-BNN). At least five different fields per condition were obtained. Quantification of signal intensity was performed using Image J software (NIH Bethesda, MD, USA).

### 2.10. Superoxide Anion Production

MAECs were incubated with PBS (control) or BPA (5 μM) for 24 h. On the day of the experiment, cells were treated with 10^−5^ M apocynin for 30 min to inhibit NADPH oxidase and then stimulated with eNOS agonists. After exposure to different experimental conditions, cells were trypsin dispersed and labeled with the DHE at 37 °C. Cells were analyzed by FACS Calibur (Becton Dickinson Company, Franklin Lakes, NJ, USA). A total of 10,000 events were analyzed for each condition.

### 2.11. Statistical Analysis

All results were expressed as mean ± standard deviation (SD). GraphPad Prism 7.0 software (GraphPad Software Inc., San Diego, CA, USA) was used for statistical analysis. First, the data distribution was analyzed using the D’Agostino-Pearson and Shapiro–Wilk normality tests. Subsequently, one-way ANOVA or Kruskal–Wallis followed by a Bonferroni or Dunns’ test, respectively, were carried out. The *p*-values presented in figures and tables correspond to the post hoc test. *p* < 0.05 was considered statistically significant.

## 3. Results

### 3.1. BPA Increases Cellular Senescence at Low Concentrations

**Figure 1:**
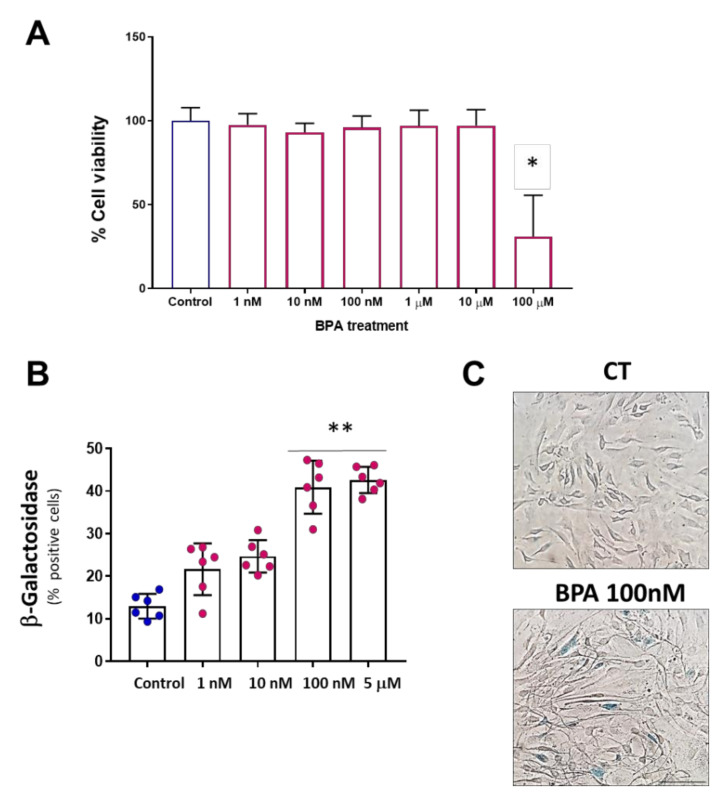
BPA induces cellular senescence at low concentrations. (**A**) MTT viability assay in MAEC treated with BPA 5 days at concentrations ranging from 1nM to 100 µM. A significant reduction in cell viability is observed at 100 µM. Data are the means ± SD (n = 3 with a triplicate per experimental condition). *p* was determined by a Kruskal–Wallis test with Dunn’s multiple comparisons test. * *p* < 0.0001. (**B**) Senescence-associated β-Gal assay in MAEC treated with BPA 5 days at the indicated concentrations. Note that the maximum effect on cell senescence was observed at concentrations of 100 nM and 5 µM. Data points represent the mean ± SD (n = 6 in triplicate). *p* was determined by a Kruskal–Wallis test with Dunn’s multiple comparisons test. ** p < 0.001. (**C**) Representative microphotographs of the senescence assay (scale bar = 50 µm). Senescent cells show the characteristic staining of β-Gal in blue color, CT means control group.

Previously, we have reported that BPA can induce a substantial reduction in cell viability at high concentrations in different cell lines, including endothelial cells at 24 h [18,20]. To test the effects on cell viability of longer BPA administration, we extended our study to 5 days. As is shown in Figure 1A, MAEC treatment with BPA did not induce any change in cell viability at BPA concentrations below 100 µM. However, it induces a significant reduction in cell viability at concentrations of 100 µM at 5 days. To test whether BPA may induce cell senescence, we performed a β-Gal assay. Five days BPA treatment, at concentrations below the cytotoxic threshold (1 nM–5 μM), induced a concentration-dependent increase in the percentage of β-Gal-positive cells. As shown in Figure 1B, the concentrations that induced significant changes were 100 nM and 5 µM, half of the highest concentration at which no effects on cell viability were observed.

**Figure 2:**
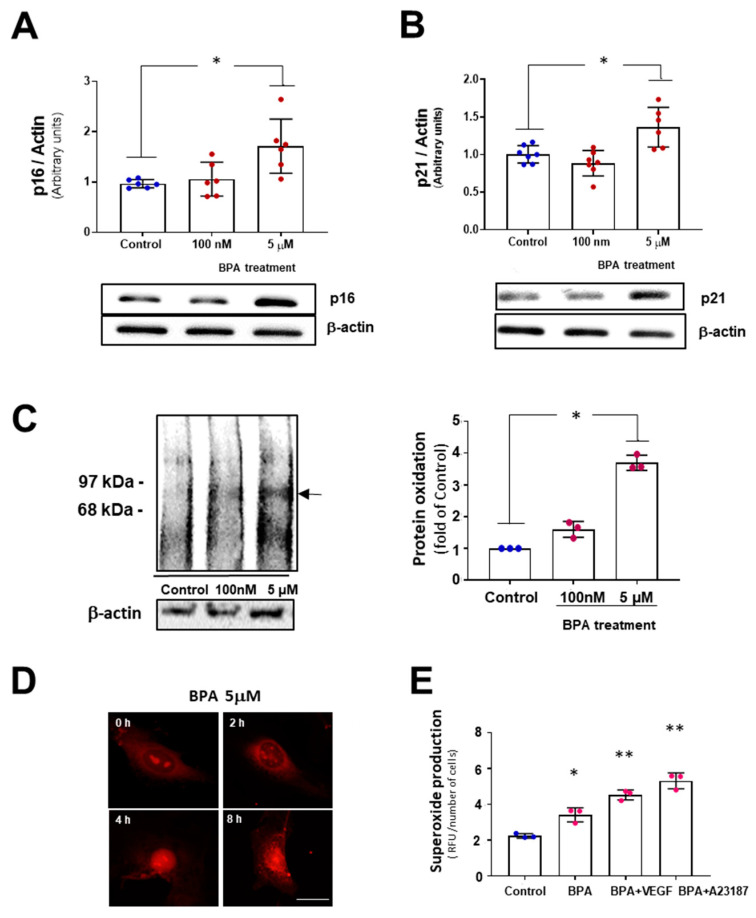
BPA induces senescent protein markers p21 and p16 and increases oxidative stress. Western blotting analysis of MAEC treated with 100 nM and 5 µM BPA for five days using antibodies against (**A**) p21 and (**B**) p16. β-actin was used as a loading control. (n = 6). Results are given as mean ± SD, *p* was determined by a Kruskal–Wallis test for the comparison between control and BPA-treated cells.* *p* < 0.05 ); (**C**) The levels of reactive oxygen species induced in MAEC by BPA were analyzed by oxyblot assay in 100 nM and 5 µM BPA-treated MAEC for 24 h to detect carbonyl groups in proteins as a marker of protein oxidation. The densities of the proteins between 97 and 68 KDa were normalized using the expression of β-actin. (n = 3 with duplicate for each condition).* *p* < 0.001 using Kruskal–Wallis test for the comparison between control and BPA-treated cells; (**D**) Immunofluorescence (IF) of superoxide production in MAEC in response to 5 µM BPA for 0–8 h, using the fluorescence probe dihydroetidium (DHE). After 4 h, fluorescence can be observed at the cell nucleus. Scale bar: 10 µm; (**E**) Quantification of superoxide in MAEC upon 5 µM BPA treatment for 24 h, BPA+VEGF and BPA+ A23187 and vehicle (control). (n = 3, per duplicate). Results are given as mean ± SD, *p* was determined by a Kruskal–Wallis test, * *p* < 0.001 vs. Control and ** *p* < 0.001 vs. BPA.

To confirm the results observed in the β-Gal functional assay, relative expression of the senescence marker proteins p21 and p16 were analyzed. We used 100 nM and 5 µM BPA concentrations, which induced maximal changes in the previous β-gal assay. After five days of BPA treatment, both p21 and p16 proteins expression increased compared with the control group only at 5 µM BPA. No detectable differences in protein expression were found at 100 nM BPA (Figure 2).

Since oxidative stress is one of the stressors capable of inducing the senescence program, we studied if oxidative stress was preceding the effects observed. BPA treatment for 24 h induced an increase in carbonylated proteins, which are surrogate markers of protein oxidation in a dose-dependent manner. Accordingly, superoxide generation as detected by DHE staining of DNA in MAEC was increased at 5 μM BPA as early as 4 h, as demonstrated by orange nuclear staining. Superoxide production in MAEC treated with BPA 5 μM for 24 h and stimulated with eNOS agonists VEGF (50 ng/mL, 3 min) and A23187 (10^−6^ M, 5 min) was quantified in the presence of an inhibitor of NADPH oxidase (apocynin, 10^−5^ M). We observed an increased production of superoxide in the presence of BPA, which was enhanced with VEGF and calcium ionophore A23187. This result suggests that eNOS uncoupling could be contributing to the increased superoxide. In summary, BPA induces oxidative stress at early time points and stimulates cellular senescence in endothelial cells.

### 3.2. BPA Modulates the PERK-ATF4-CHOP Pathway after Five Days Treatment

**Figure 3:**
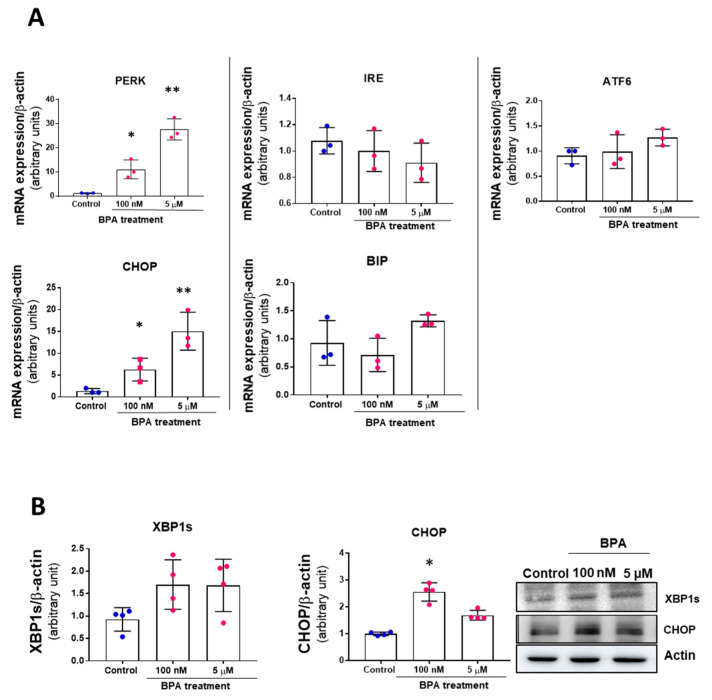
BPA induces the PERK/ATF4/CHOP branch of the UPR. (**A**) RT qPCR of control and 5 days BPA MAEC showing endothelial mRNA expression of PERK, IRE, ATF6, CHOP, and BIP. mRNA expression was normalized by β-actin. (**B**) MAEC were treated with 100 nM and 5 µM BPA for five days, and XBP-1 spliced form (XBP-1s) indicative of IRE1α activation, and CHOP protein expressions were measured by Western blot. β-actin was used as a loading control. Data represent media + SD of 4 independent experiments, each performed in duplicate. * *p* < 0.05 and ** *p* < 0.01 using Kruskal–Wallis test for the comparison between control and BPA-treated cells.

Since the UPR is known to be part of the senescent phenotype, we next explored whether UPR is involved in BPA responses in MAEC. As shown in Figure 3A, BPA treatment induced an increase in the PERK-ATF4-CHOP pathway. Slight increases in BIP and ATF6 mRNAs expression are observed at 5 µM, although they were not statistically significant. In addition, the relative expression of two essential proteins of the UPR system, XBP1 and CHOP, was also analyzed (Figure 3B). On the one hand, the IRE-XBP1 signaling pathway did not show significant changes. On the other hand, the CHOP pathway only showed significant changes at 100 nM.

Thus, only the PERK-ATF4-CHOP branch of UPR is activated in the vascular endothelial cells after BPA treatment and may contribute to the senescent phenotype observed.

### 3.3. BPA Induces Senescence and Activation of the PERK-ATF4-CHOP Pathway in Mice

**Figure 4:**
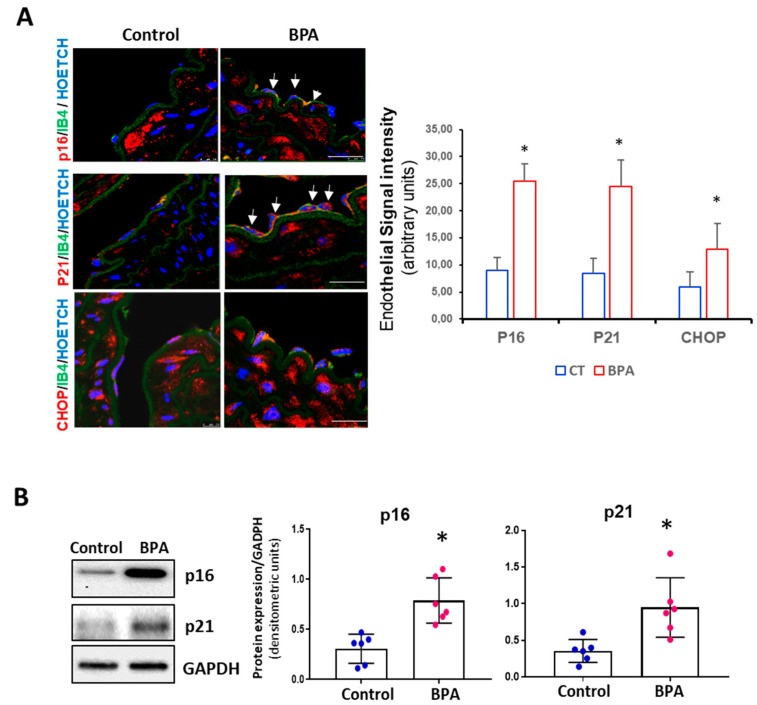
Senescence proteins (p16 and p21) and CHOP expression in BPA-treated-mice aort. Treatment with BPA in drinking water induced notable increases in expression of both the p16, p21, and CHOP proteins. (**A**) Representative confocal images from aorta sections of control and 8-weeks-treated BPA mice followed by immunostaining for p16 (upper panel), p21 (middle panel), and CHOP (lower panel) (red). Endothelium was marked in green with isolectin B4-FITC. Nuclei were labeled with hoechst in blue (n = 8 mice per condition). Scale bar = 60 µM. (**B**) Immunoblot detection of p16 and p21, in total aorta lysates from CT and 8 weeks BPA-treated mice. GAPDH was used as a loading control. A representative immunoblot is shown. The densitometric analysis is shown below (data are mean ± SD, n = 6 mice per condition) * *p* < 0.001 vs. CT.

To test the potential of BPA to trigger senescence in vivo, 3-month-old CD1 mice were administered BPA in the drinking water for eight weeks. p16 and p21, the main proteins involved in stress-associated cell senescence, were overexpressed in the endothelium of animals treated with BPA compared to the control mice (Figure 4A). In addition, there was also an increase in CHOP expression, confirming the participation of this pathway in the senescent response to BPA. Next, we examined the expression of senescence proteins p16 and p21 in the aorta by Western blot. Quantitative analysis showed a significant increase in p16 and p21 levels in BPA-treated mice compared to control (Figure 4B).

### 3.4. NAC Reduces Cell Senescence and Attenuates the BPA-Induced Response on the UPR System

**Figure 5:**
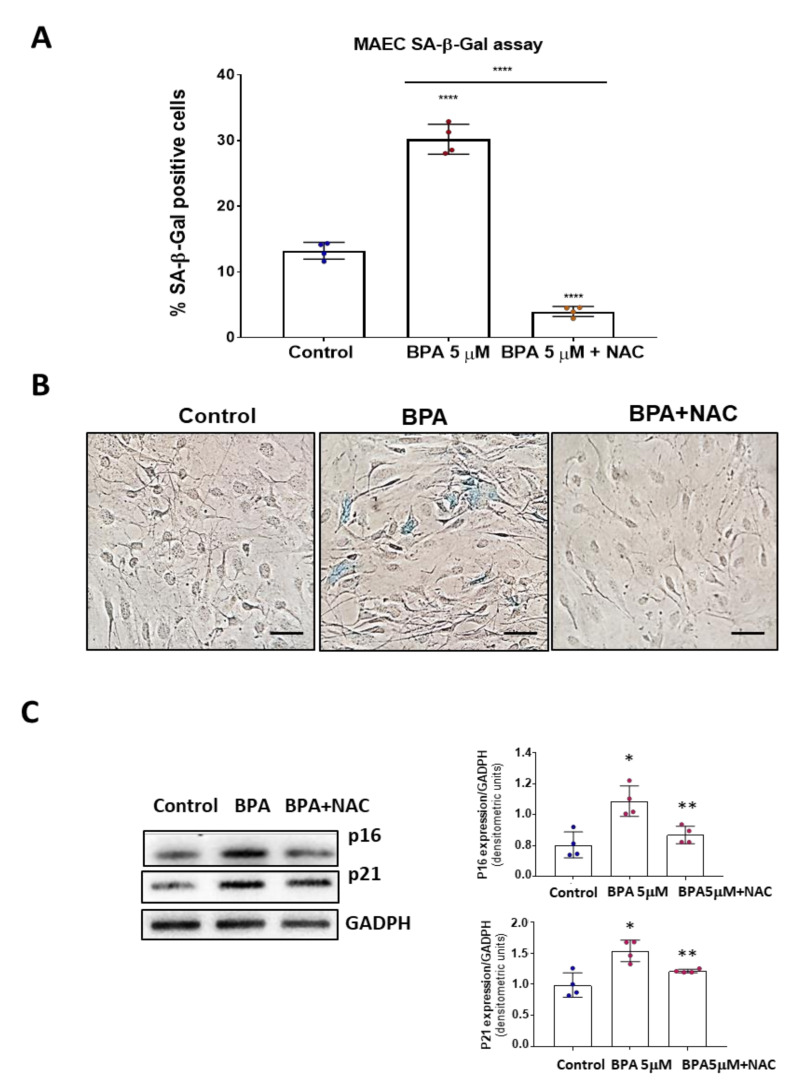
N-Acetylcistein decreases senescence-associated β-Gal assay in MAEC treated with BPA 5 days. (**A**) Senescence-associated β-Gal assay in MAEC treated with BPA 5 days at 5 µM and BPA 5 µM + NAC 5 mM. Note that pretreatment with NAC in BPA-treated cells reduced the percentage of senescent cells even below the control group. Results represented are means ± SD. (n = 4, each performed in triplicate). **** *p* < 0.0001 using Kruskal–Wallis test. (**B**) Representative microphotographs of the senescence assay made at x40 magnification. (**C**) Western blot analysis of MAEC treated as in **A**, using antibodies to p21 and p16. Data are the means ± SD of four different experiments, each performed in duplicate. * *p* < 0.05 using Kruskal–Wallis test for the comparison between control and BPA-treated cells; ** *p* < 0.001 using Kruskal–Wallis test for the comparison between BPA 5 µM and BPA 5 µM + NAC 5 mM.

Since BPA can lead to cell senescence in vivo and cultured endothelial cells and also induce oxidative stress, a known inductor of cell senescence, we wonder whether an antioxidant could revert its effects. Thus, we tested whether a general antioxidant drug, N-acetylcysteine (NAC), could ameliorate BPA effects on MAEC. We found that NAC co-treatment reversed BPA effects on endothelial cell senescence. Indeed, β-Gal assays in MAEC treated 5 days with BPA or BPA+ NAC showed that NAC not only prevented the senescence triggered by BPA but reduced the number of senescence cells below those in the control group (Figure 5A). Accordingly, p16 and p21 protein expression was also reduced in the presence of NAC (Figure 5B). These results suggest a causative role of BPA-induced oxidative stress in cellular senescence.

**Figure 6:**
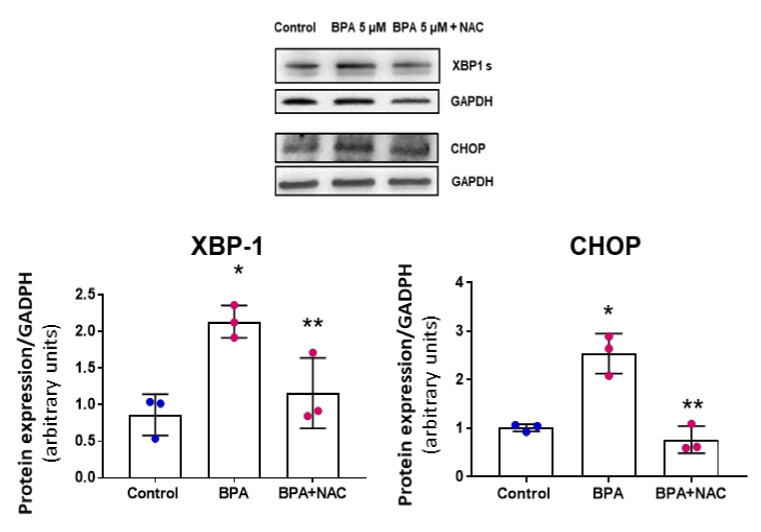
NAC prevents early UPR activation induced by BPA treatment in MAEC. A. Western blotting analysis of MAEC treated with 5 µM BPA and BPA 5 µM + NAC 5 mM for 24 h using antibodies against XBP1 and CHOP. There was a significant increase in XBP-1 and CHOP, which could be avoided by co-treatment with NAC. Results represented are means ± SD. (n = 3, each performed in duplicate). * *p* < 0.0001 using Kruskal–Wallis test for the comparison between control and BPA-treated cells ** *p* < 0.0001 using Kruskal–Wallis test for the comparison between BPA and BPA + NAC.

Next, we tested if NAC is able to prevent also the UPR response. Western blots were performed to analyze the relative expression of the UPR proteins CHOP and XBP1 at 24 h. A significant increase in the expression of the XBP1 and CHOP proteins was observed in response to 24 h treatment with BPA, and NAC reversed the BPA effect. Thus, increased oxidative stress and oxidation of proteins lead to early UPR response, which can be inhibited by NAC (Figure 6).

## 4. Discussion

Our results show, for the first time, that BPA in doses under the cytotoxic threshold can induce accelerated cell aging in mouse aortic endothelium. Our results suggest a causative role of BPA-induced oxidative stress in cellular senescence and point to UPR as a mediator in this process.

It is widely accepted that during aging exists a poor cellular stress response contributing to age-related diseases [63]. Recently, it has been demonstrated that the accumulating population of senescent cells in the aged human organism indeed experience proteostasis decline, failing to properly activate multiple programs required for stress adaptation at the level of gene transcription, including the UPR. Our results demonstrate that BPA, a ubiquitous contaminant, at low doses can activate an endothelial senescence program in cultured cells and, more importantly, in vivo, inducing a state of accelerated cellular aging.

The viability assays showed cytotoxic effects of BPA at the high micromolar concentration range. This range is consistent with the results described by our group in human podocytes [20], endothelial cells [19], or by other researchers in different cell lines [64,65,66]. Subsequent experiments were planned, using a range of concentrations that oscillated from 1 nM to 5 µM (half of the highest concentration at which no effects on cell viability were observed).

We observed at the selected concentrations that BPA induces senescence in MAEC. There is strong evidence that BPA is capable of inducing senescence in other cell lines and animal models. Mahmudi et al. [28] observed that BPA could induce senescence in human fetal lung fibroblasts through the ATM-p53 signaling pathway. Ribeiro-Varandas et al. [29] observed differences in p21 gene expression at the vascular level as a function of cell age in HUVEC treated with one µg/mL of BPA. Furthermore, Soundararajan et al. [30] observed that BPA could increase the transcription of senescence markers in zebrafish embryos in a situation of metabolic stress such as hyperglycemia. The same group observed in patients with type 2 diabetes a positive correlation between systemic levels of BPA and senescence markers such as p16, p21, p53, and GLB1 [31]. The two main effectors activated in response to different senescence-inducing stimuli are p16 and p21 [67]. In our study, five-day treatment with BPA induced significant changes in β-Gal activity at 100 nM and 5 µM, although, in the Western blot analysis, p16 and p21 protein expression was only increased at the higher concentration used. Finally, exposure to BPA has been correlated with obesity-associated breast cancer since it can positively regulate human telomerase reverse transcriptase [68]. In human studies, senescence markers (p21, p16, and p53) have been associated with age and a sedentary lifestyle. Interestingly, physically active old people did not show significant differences with young people [67]. Similarly, significant increases in senescence proteins have been observed in endothelial cells of aged sedentary individuals vs. aged exercising adults [24]. In fact, senescent cells are considered a therapeutic target to treat cardiovascular diseases, and several trials with senolytic drugs are in process [69].

Senescence can be induced by various types of stress, including oxidative stress [70]. Our results demonstrate that BPA exposure even at 100 nM concentrations can increase superoxide production, lipid peroxidation, and protein oxidation in 24 h. Oxidative stress can trigger ER stress, which can induce the UPR pathway. In addition, senescent cells may also contribute to ER stress releasing inflammatory cytokines, growth factors, and enzymes involved in extracellular matrix remodeling. For those reasons, senescence can be considered an adaptive stress response. It has been demonstrated that ER stress/UPR activation occurs at senescence [41]. Evidence suggests that the UPR system can induce the senescent phenotype. However, other authors suggest that the UPR is part of the senescent phenotype because senescence can induce the activation of the UPR system [53]. Our results show that 5 μM BPA treatment increases p16 and p21 proteins levels, which could be related to the significant increase in PERK and CHOP mRNAs. Furthermore, it is noteworthy that after prolonged exposure over time, both in cell cultures and in the animal model, overexpression of the PERK-ATF4-CHOP pathway occurs.

Interestingly, although most studies related the CHOP signaling pathway to death processes in cell cultures [49], others have observed that it regulates or activates cellular aging processes and senescence, independently of cell death [53]. A recent article highlighted that all arms of the UPR were activated and associated with replicative senescence in WI38 cells, and in H2O2-induced senescence, the same cells, only the PERK branch was activated [71]. In addition, this signaling pathway has also been associated with myocardial ischemia, hypertension, cardiac atrophy, hypertrophy, or even vascular calcification [43,44,45,46,47].

Furthermore, our previous studies report that BPA can induce RIP3-necroptosis both in vivo and in endothelial cells [19]. We observed increased necroptosis in MAEC 24 h after BPA treatment at 10 μM, while no increase in apoptotic cell death was detected. However, the percentage of cells undergoing necroptotic cell death was modest. It is accepted that overwhelming stress will cause cell death, while less intense stress will cause senescence [68]. In the present study, BPA needed 5 days to show a significant increase in senescence markers. Thus, it is possible that in the short term, BPA induces necroptosis in endothelial cells, but longer treatments may lead to cellular senescence.

Several experiments show that blocking the PERK pathway can modify the percentage of SA-β-Gal-positive cells in different cell models [53]. However, the mechanisms of interaction between senescence proteins and those of the UPR system remain unclear. Our 24-h protein expression studies showed an increase in protein expression in two different pathways of the UPR system, and co-treatment with NAC prevented the overexpression of XBP-1 and CHOP proteins. NAC treatment reduced cellular senescence at 5 days even below the control group, indicating a temporal pattern in the endothelial responses to low concentrations of BPA. In our experiments, oxidative stress and UPR lead to senescence.

In epidemiological studies, exposure to BPA has been positively correlated with the predisposition to develop cardiovascular diseases. For example, Shankar et al. [22] observed a positive correlation between hypertension and increasing urinary BPA levels, independent of other factors such as age, sex, or ethnicity. Similarly, Aekplakorn et al. [23] demonstrated a similar relationship in a Thai cohort. Finally, the work of Bae et al. [72] showed that people who drank canned beverages had higher urinary BPA levels than those who drank from glass containers, and those with higher BPA levels had a significant increase in blood pressure.

Today there is still controversy about the degree of exposure to BPA and the doses considered safe. In a recent analysis of our group, it was determined that the mean urinary concentration of BPA in the general population is close to 10 nM, which may be related to ingested concentrations close to the doses used in our study [73]. The doses used in the present work, 100 nM and 5 µM, have been detected in certain groups with high exposure, such as workers in the plastic industry [74,75,76,77]. Other population groups in which unusually high concentrations have been detected are patients with stage 5 chronic kidney disease undergoing hemodialysis or patients in intensive care units [78,79]. On the other hand, the dose administered to animals is half the dose used by the European Food Safety Authority as a reference to calculate the human tolerable daily intake [1]. Furthermore, the U.S. National Toxicology Program defines doses less than 50 mg/kg/day as “low dose” [57]. Furthermore, in the CLARITY-BPA study (Consortium Linking Academic and Regulatory Insights on Bisphenol A Toxicity), one of the largest animal studies conducted in the context of BPA, the authors stated, “BPA did not elicit adverse effects in the in-life or terminal endpoints monitored in either sex below 25,000 μg/kg bw/day” [58]. However, other authors suggest that some results described in this study show non-monotonic effects, reaffirming the need to study the effects of BPA at low doses [59]. In our “low-dose” animal model, BPA visibly affected the vasculature of the exposed animals, promoting the overexpression of the proteins p16, p21, and CHOP. These data confirm the results described in the cell model, showing that BPA can promote accelerated cellular aging.

Thus, in line with the data extracted from the academic literature, BPA is an environmental factor involved in accelerated cell aging mechanisms, affecting the vascular endothelium and promoting cardiovascular diseases.

## Data Availability

Data is contained within the article.
